# Enhanced etoposide sensitivity following adenovirus-mediated human topoisomerase II **α** gene transfer is independent of topoisomerase II **β**

**DOI:** 10.1054/bjoc.2001.1966

**Published:** 2001-09

**Authors:** Z Zhou, L A Zwelling, R Ganapathi, E S Kleinerman

**Affiliations:** Department of Cancer Biology; Department of Pediatrics; Division of Medicine, The University of Texas MD, Anderson Cancer Center, Box 173, 1515 Holcombe Boulevard, Houston, 77030, Texas; The Cleveland Clinic, Cleveland, Ohio, 44195, USA

**Keywords:** topoisomerase IIα, topoisomerase IIβ, etoposide, amsacrine, drug targetting

## Abstract

The roles that the α and β isoforms of topoisomerase II (topo II) play in anticancer drug action were determined using MDA-VP etoposide-resistant human breast cancer cells and a newly constructed adenoviral vector containing the topo IIα gene (Ad-topo IIα). MDA-VP cells were more resistant to etoposide than to amsacrine and had more resistance to etoposide than did MDA-parental cells. MDA-VP cells also expressed lower topo IIα RNA and protein levels than parental cells but had comparable topo IIβ levels. After infection with Ad-topo IIα, topo IIα, RNA and protein levels increased significantly, as did the cells' sensitivity to etoposide. In contrast, topo IIβ levels remained constant with little alteration in the cells' sensitivity to amsacrine. Band-depletion immunoblotting assays indicated that topo IIα was depleted in etoposide-treated, Ad-topo IIα-transduced MDA-VP cells but not in amsacrine-treated cells. Topo IIβ was depleted in amsacrine-treated, Ad-topo IIα-MDA-VP cells, with little change in the topo IIα levels. These results suggest that topo IIα gene transfer does not alter topo IIβ expression and that enhanced sensitivity to etoposide is therefore secondary to change in topo IIα levels. These studies support the theory that etoposide preferentially targets topo IIα, while amsacrine targets topo IIβ. © 2001 Cancer Research Campaign http://www.bjcancer.com


BJOC 01-1966 747-751 20/8/01 3:29 pm Page 747

*British Journal of Cancer* (2001) **85**(5), 747–751
© 2001 Cancer Research Campaign
doi: 10.1054/ bjoc.2001.1966, available online at http://www.idealibrary.com on IDEAL
http://www.bjcancer.com

### Enhanced etoposide sensitivity following adenovirus-mediated human topoisomerase IIα gene transfer is independent of topoisomerase IIβ

**Z Zhou[1], LA Zwelling[3], R Ganapathi[4] and ES Kleinerman[2]**

[1]Department of Cancer Biology; [2]Department of Pediatrics; [3]Division of Medicine, The University of Texas MD, Anderson Cancer Center, Box 173, 1515 Holcombe Boulevard, Houston, Texas 77030; [4]The Cleveland Clinic, Cleveland, Ohio 44195, USA

**Summary** The roles that the α and β isoforms of topoisomerase II (topo II) play in anticancer drug action were determined using MDA-VP etoposide-resistant human breast cancer cells and a newly constructed adenoviral vector containing the topo IIα gene (Ad-topo IIα). MDA-VP cells were more resistant to etoposide than to amsacrine and had more resistance to etoposide than did MDA-parental cells. MDA-VP cells also expressed lower topo IIα RNA and protein levels than parental cells but had comparable topo IIβ levels. After infection with Ad-topo IIα, topo IIα, RNA and protein levels increased significantly, as did the cells' sensitivity to etoposide. In contrast, topo IIβ levels remained constant with little alteration in the cells' sensitivity to amsacrine. Band-depletion immunoblotting assays indicated that topo IIα was depleted in etoposide-treated, Ad-topo IIα-transduced MDA-VP cells but not in amsacrine-treated cells. Topo IIβ was depleted in amsacrine-treated, Ad-topo IIα-MDA-VP cells, with little change in the topo IIα levels. These results suggest that topo IIα gene transfer does not alter topo IIβ expression and that enhanced sensitivity to etoposide is therefore secondary to change in topo IIα levels. These studies support the theory that etoposide preferentially targets topo IIα, while amsacrine targets topo IIβ. © 2001 Cancer Research Campaign http://www.bjcancer.com

**Keywords**: topoisomerase IIα, topoisomerase IIβ, etoposide, amsacrine, drug targetting

DNA topoisomerase II (topo II) is a target for many anticancer drugs, including etoposide and amsacrine. These drugs stabilize the topo II-DNA cleavable complex, preventing religation of the DNA strand. Breaking of double-stranded DNA subsequently leads to cell death (Malonne and Atassi, 1997). Two distinct topo II isoforms have been identified. The topo IIα gene, located on chromosome 17q21–22 encodes a 170-kDa enzyme, and the Topo IIβ gene located on chromosome 3p24, encodes a 180-kDa enzyme (Tsai-Pflugfeder et al, 1988; Jenkins et al, 1992). These two isoforms have different functions in DNA topography and the cell cycle (Austin and Marsh, 1998; Tan et al, 1992). However, both enzymes have been implicated in topo II-reactive drug action. Anticancer drug resistance has been attributed to alteration of topo II gene expression. Determining the importance of each isoform in anticancer drug action and resistance may create novel approaches to circumventing drug resistance and screening new isoform-specific drugs. Initial studies will require cell lines that either lack or express low levels of one of the topo II isoforms. We have previously shown that MDA-VP etoposide-resistant human breast cancer cells express low levels of topo IIα compared MDA parental cells (Zhou et al, 1999). Here we show that MDA-VP and parental cells have comparable topo IIβ levels. MDA-VP cells therefore provide a useful model to study the role of each topo II isoform in drug sensitivity. The newly constructed adenovirus vector containing the human topo IIα gene (Ad-topo IIα) has made it possible to sensitize cells to topo IIα-reactive drugs. Our present study indicates that etoposide preferentially interacts with the topo IIα isoform, while topo IIβ is the preferred target for amsacrine. These results confirm previously reported studies on the interaction between the two topo II isoforms and topo II-targetting drugs (Errington et al, 1999).

*Received 15 November 2000*
*Revised 4 May 2001*
*Accepted 17 May 2001*

*Correspondence to:* ES Kleinerman

### MATERIAL AND METHODS

### Cell lines

MDA-MB-231 parental cells were obtained from American Type Culture Collection (Manassas, VA, USA). MDA-VP etoposide-resistant human breast cancer cells were initially derived and cloned from MDA-parental cells as described previously (Matsumoto et al, 1997). All cells were screened and found to be free of *Mycoplasma* (Gen-Probe Co., San Diego, CA, USA).

### Infection of cells with Ad-topo IIα virus

The Ad-topo IIα virus was constructed and purified as described previously (Zhou et al, 1999). Cells were grown in logarithmic phase and were infected with Ad-topo IIα at a multiplicity of infection of 100 pfu cell. Cells were harvested by standard methods after 48 h.

### Cytostasis assay

A total of 5000 cells were seeded onto 96-well cell culture plates and allowed to adhere overnight. Cells were treated with different concentrations of etoposide or amsacrine (Sigma Co., St Louis, MO, USA). Their antiproliferative activity was determined by the

3-[4,5-dimethylthiazol-2-yl]-2,5-diphenyltetrazolium bromide; Thiazolyl blue (MTT) assay as described previously (Fan et al, 1994).

### Northern blot analysis

Total RNA was extracted with Trizol Reagent (Life Technologies, Inc., Grand Island, NY, USA). Then 20 μg of RNA was electrophoresed on a 1% formaldehyde/agarose gel and transferred to a Hybond-$N^+$ membrane (Amersham Corp., Arlington Heights, IL, USA). Human topo IIα gene probe ZII69 (Tsai-Pflugfeder et al, 1988), topo IIβ gene probe F12 (Austin et al, 1993; Herzog et al, 1998), and a GAPDH probe were used for hybridization. Probes were labelled using the Rediprime labelling system (Amersham).

### Western blot analysis

The procedure involved 2 million cells, set up in a 100 mm dish, and treated as indicated. Cells were washed with cold phosphate-buffered saline (PBS) and lysed with buffer (50 mM Tris-HCl, pH 8.0, 425 mM NaCl, 1% Nonidet P-40, 0.5% deoxyclolate, 0.1% SDS, 10 mM β-mercaptoethanol) containing protease inhibitors. (Ganapathi et al, 1996) Then 50 μg protein was run on a 7.5% SDS/polyacrylamide gel and transferred to a Hybond ECL nitrocellulose membrane (Amersham). Human topo IIα antibody (TopoGEN, Inc., Columbus, OH, USA), topo IIβ antibody (PharMingen Inc., San Diego, CA, USA), and β-actin antibody were used for protein detection with the ECL analysis system (Amersham).

### Band-depletion immunoblotting assay

The band-depletion immunoblotting assay was performed as described previously (Zwelling et al, 1989). Cells were infected with Ad-topo IIα or Ad-β-gal (control) for 48 h and then treated with 200 μM etoposide or 100 μM amsacrine at 37 °C for 1 h as indicated. Cell lysates were prepared in 2X Laemmli buffer by sonication for 30 s and boiled for 5 min. Proteins were resolved on a 7.5% SDS/polyacrylamide gel and immunoblotted using human topo IIα, topo IIβ, and β-actin antibodies.

**Table 1** Comparison of resistance to etoposide or amsacrine between MDA-parent cells and MDA-VP cells.*

| **Cells** | **$IC_{50}$ † for etoposide (M ± SD)‡** | **$IC_{50}$ for amsacrine (M ± SD)‡** |
|---|---|---|
| MDA-parental cells | 3.0 ± 0.2 μM | 5.6 ± 0.3 μM |
| MDA-VP cells | 45.6 ± 2.0 μM | 12.8 ± 1.0 μM |
| **Relative resistance of MDA-VP cells §** | **15.0-fold** | **2.2-fold** |

*Cytostasis was measured by MTT assay as described in Material and methods. †$IC_{50}$ is the concentration that inhibits 50% of cell growth. ‡M: mean value from at least 3 experiments, SD: standard deviation. §The relative resistance is calculated by dividing the $IC_{50}$ of MDA-VP by $IC_{50}$ of MDA-parent cells.

### RESULTS

MDA-VP and parental cells were treated with various concentrations of etoposide or amsacrine. Table 1 shows that MDA-VP cells were 15-fold more resistant to etoposide than were MDA-parental cells. In contrast, MDA-VP cells were only 2.2-fold more resistant to amsacrine. To determine whether the differences in resistance were related to expression of the topo II isoforms in these cells, topo IIα and topo IIβ RNA and protein levels were measured.

Topo IIα mRNA levels were lower in MDA-VP cells than in MDA-parental cells (Figure 1A). Densitometric analysis showed only 20% topo IIα gene expression in the etoposide-resistant MDA-VP cells compared to the MDA-parent cells (Figure 1B,

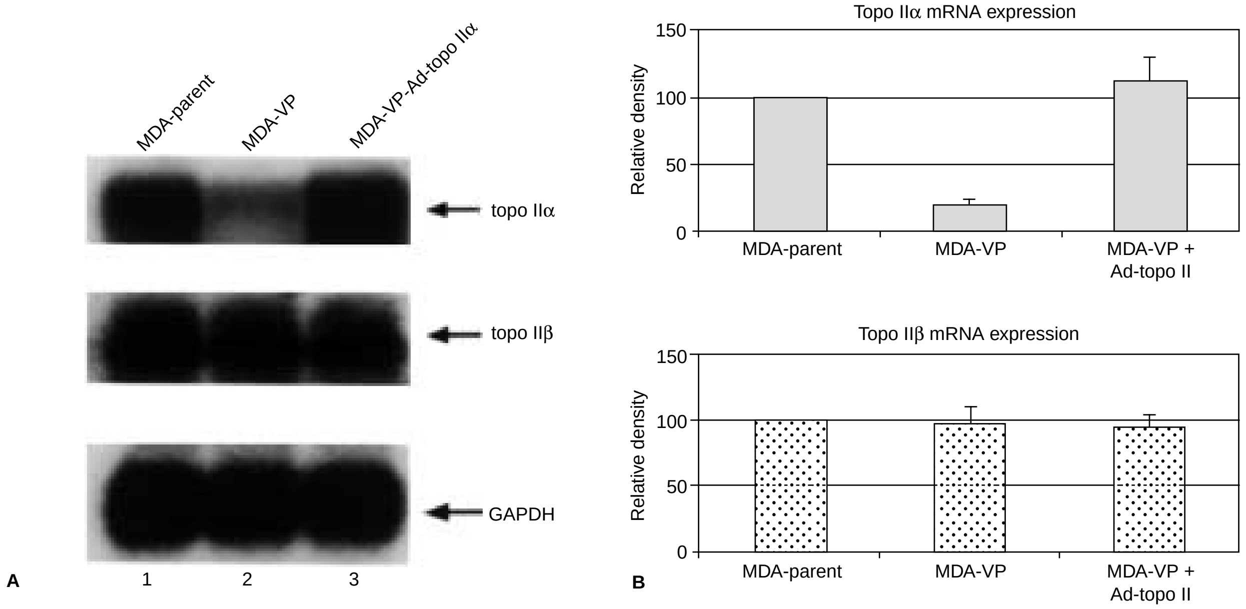

**Figure 1** (**A**) Expression of topo IIα and topo IIβ RNA in MDA-parental cells, MDA-VP cells, and MDA-VP cells infected with Ad-topo IIα. Total RNA was extracted from MDA-parental cells (lane 1), MDA-VP cells, (lane 2) and MDA-VP cells infected with Ad-topo IIα at 100 pfu/cell (lane 3). Northern blot analysis was performed using topo IIα, topo IIβ and GAPDH probes. (**B**) Topo IIα and β mRNA expression was quantified using densitometric analysis. The relative density at each point was calculated by dividing that value by the density in MDA-parent cells and adjusted by GAPDH loading control. The columns represent the mean from 3 independent experiments; the bars represent the standard deviation

 *© 2001 Cancer Research Campaign*

BJOC 01-1966 747-751 20/8/01 3:29 pm Page 749

upper panel). Topo IIβ mRNA levels were not significantly different in the two cell types (Figure 1B, lower panel). Topo IIα protein levels were also much lower in MDA-VP cells than in MDA-parental cells, (Figure 2A and 2B, upper panel) while topo IIβ protein levels were *not* significantly different in the 2 cell types (Figure 2B, lower panel).

To further explore the relationship between drug resistance and isoform expression, MDA-VP cells were infected with the Ad-topo IIα virus, then the topo II RNA and protein levels, as well as drug sensitivity, were quantified. Topo IIα mRNA levels were elevated after infection; however, topo IIβ levels were not significantly altered (Figure 1). Topo IIα protein levels also increased in MDA-VP cells after Ad-topo IIα infection, but topo IIβ protein levels remained constant (Figure 2). The sensitivity of MDA-VP cells to etoposide increased 4.5-fold after infection with Ad-topo IIα. The $IC_{50}$ of MDA-VP cells infected with Ad-topo IIα went from 45.6 μM to 10.1 μM. By contrast, the sensitivity to amsacrine only increased 1.3-fold following infection with Ad-topo IIα (Table 2). Therefore, the increased sensitivity of cells to etoposide following topo IIα gene transfer correlated with increased topo IIα levels but not with topo IIβ levels.

A band-depletion immunoblotting assay was performed with topo IIα and topo IIβ antibodies to analyze the interaction of the 2 isoforms with etoposide and amsacrine. Topo IIα band depletion was seen following treatment with etoposide, while little change was seen following amsacrine treatment (Figure 3, lanes 2,3, upper panel). In contrast, topo IIβ was more depleted in cells treated with amsacrine than in cells treated with etoposide (Figure 3, lanes 2,3, middle panel). Densitometric analysis indicated that etoposide induced 70% depletion of topo IIα protein and only 10% depletion of topo IIβ protein (Figure 3, lane 2). Conversely, amsacrine induced only a 10% reduction of topo IIα protein, but a 60% reduction of topo IIβ protein (Figure 3, lane 3). After infection of MDA-VP cells with Ad-topo IIα, topo IIα protein levels were once again significantly increased (Figure 3, lane 4, upper panel) with relatively no change in topo IIβ protein levels (Figure 3, lane 4, middle panel). Neither topo IIα protein levels nor topo IIβ protein levels were significantly altered following infection with Ad-β-gal (Figure 3, lane 7). The band-depletion pattern in MDA-VP cells following infection with Ad-topo IIα (Figure 3, lanes 5, 6) and Ad-β-gal (Figure 3, lanes 8, 9) was the same as that seen in MDA-VP control cells (Figure 3, lanes 2,3). Etoposide treatment induced a significant reduction in topo IIα, with little change in topo IIβ. By contrast, treatment with amsacrine did not affect the increased topo IIα protein levels in the MDA-VP-Ad-topo IIα cells.

**Table 2** Enhancement of sensitivity to etoposide or amsacrine following infection of MDA-VP cells with Ad-topo IIα*

| **Cells** | **$IC_{50}$ for etoposide (M ± SD)** | **$IC_{50}$ for amsacrine (M ± SD)** |
|---|---|---|
| MDA-VP cells | 45.6 ± 2.0 μM | 12.8 ± 1.0 μM |
| MDA-VP cells infected with Ad-topo IIα | 10.1 ± 0.5 μM | 9.3 ± 0.4 μM |
| **Sensitivity enhancement**† | **4.5-fold** | **1.3-fold** |

*MDA-VP cells were infected with Ad-topo IIα (100 pfu/cell) for 48 h, then treated with different concentrations of etoposide or amsacrine. †Sensitivity enhancement is calculated by dividing the $IC_{50}$ of MDA-VP by $IC_{50}$ of MDA-VP with Ad-topo IIα.

### DISCUSSION

Topo II, a nuclear enzyme involved in a number of important cellular processes, is the target for several anticancer drugs. The specific roles of topo IIα and topo IIβ isoforms in the action of these topo II-targetting drugs are still poorly understood. Our data provide further evidence that topo IIα is the main target for etopo-

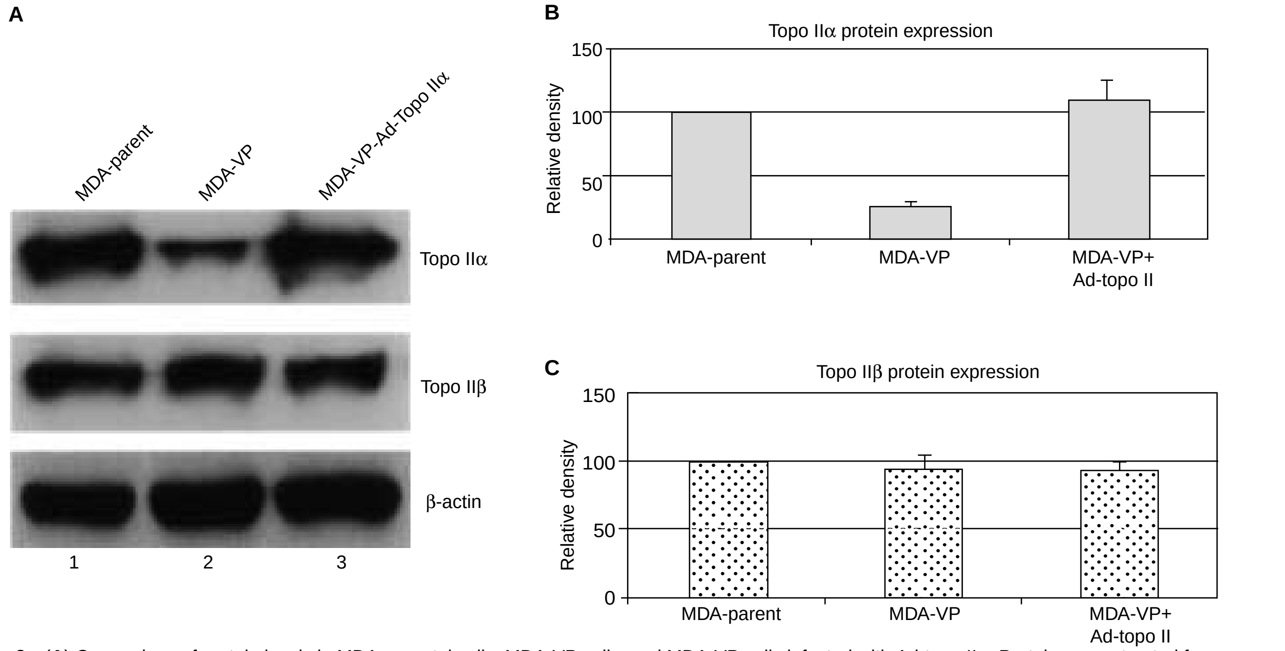

**Figure 2** (**A**) Comparison of protein levels in MDA-parental cells, MDA-VP cells, and MDA-VP cells infected with Ad-topo IIα. Protein was extracted from MDA-parental cells (lane 1), MDA-VP cells (lane 2), and MDA-VP cells infected with Ad-topo IIα (lane 3). Western blot analysis was performed using anti-human topo IIα, topo IIβ, and β-actin antibodies. (**B**) Topo IIα and β protein expression was quantified using densitometric analysis. The relative density was calculated compared with MDA-parent cells and adjusted by β-actin loading control. The columns represent the mean from 3 independent experiments; the bars represent the standard deviation

*© 2001 Cancer Research Campaign* 

BJOC 01-1966 747-751 20/8/01 3:29 pm Page 750

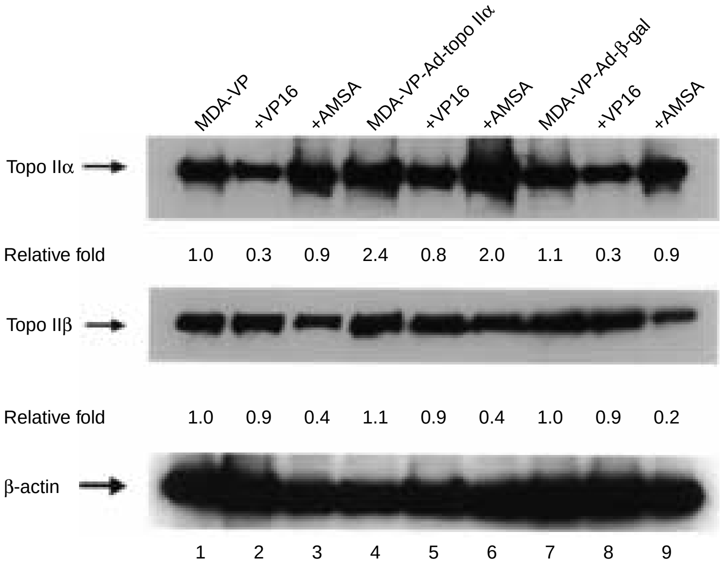

**Figure 3** Band-depletion immunoblotting assay using topo IIα and topo IIβ antibodies. MDA-VP cells were treated with PBS buffer as a control (MDA-VP), with 200 μM etoposide (VP16), or with 100 μM amsacrine (AMSA) for 1 h at 37˚C. Identical treatment was performed on MDA-VP cells 48 h after infection with Ad-topo IIα (lanes 4–6) or Ad-β-gal (lanes 7–9). Topo II protein isoforms were extracted and quantified using a band-depletion immunoblotting assay with human topo IIα, topo IIβ, and β-actin antibodies. The relative fold was calculated using densitometric analysis from 3 independent experiments. MDA-VP cells were designated as 1.0 and calculations were adjusted according to the β-actin protein loading control side, while topo IIβ is the preferred target for amsacrine in MDA-VP cells. The etoposide sensitivity and resistance are more related to topo IIα gene expression than to topo IIβ expression. MDA-VP cells expressed lower levels of topo IIα RNA and protein than MDA parental cells. In contrast, topo IIβ RNA and protein levels were relatively the same in both cell types. MDA-VP cells are more resistant to etoposide than to amsacrine and this correlates to the topo IIα and topo IIβ protein levels. Correlation between mRNA topo II levels and cell kill are not always universal. The level of drug-stabilized cleavable complex formation is the most important factor (Koo et al, 1999). Our previous studies show that etoposide-induced topo IIα-DNA cleavable complex formation is also significantly lower in MDA-VP cells than in parental cells, supporting the hypothesis that low levels of topo IIα account for the etoposide resistance of these cells. Drug uptake and participation of P-glycoprotein or the multiple drug-resistant associated protein do not play a role in resistance of MDA-VP cells (Asano et al 1996).

Transfer of the human topo IIα gene into MDA-VP cells using an adenoviral vector increased topo IIα protein levels without an appreciable change in topo IIβ protein levels. The topo IIα protein produced following transduction was sensitive to etoposide but not to amsacrine. Etoposide-induced cytotoxicity was enhanced 4.5-fold in cells transduced with topo IIα, whereas amsacrine-induced cytotoxicity did not change significantly. These results indicate that topo IIα gene transfer does not alter topo IIβ expression and that the enhanced sensitivity to etoposide is secondary to the change in topo IIα.

The involvement of topo IIβ in amsacrine sensitivity is also supported by others. Herzog et al (1998) have shown that topo IIβ mRNA levels in HL60/AMSA amsacrine-resistant human leukaemia cells are only 10% of those in HL-60 parental cells and that topo IIβ protein is not detectable in HL60/AMSA cells. However, these cells are sensitive to etoposide. Withoff et al (1996) have additionally demonstrated that amsacrine resistance in GLCA/AM3y cells, a subline of the human small cell lung carcinoma cell line, is linked to a major decrease in topo IIβ protein. Dereuddre et al were able to increase the sensitivity of a Chinese hamster lung cell line to amsacrine by transfection with the topo IIβ gene (Dereuddre et al, 1997).

Topo IIβ have different tissue distribution. High levels of topo IIα expression have been seen in aggressive proliferating tumours, whereas topo IIβ appears to be expressed ubiquitously in quiescent cells (Turley et al, 1997). Topo IIα is essential for survival of eucaryotic cells (Wang, 1996), while topo IIβ does not appear to be essential for either proliferation or survival (Yang et al, 2000; Herzog et al, 1998). Such findings may help explain the greater clinical utility of etoposide versus amsacrine. Each topo II isoform appears to carry out a different cellular function and plays a different role in drug resistance. It is important to understand how tumour cell sensitivity may be influenced by differential expression of these two isoforms.

In summary, our data indicate that topo IIα gene transfer does not affect topo IIβ expression, and the ability to circumvent etoposide resistance using topo IIα gene transfer is secondary to enhanced production of the drug-sensitive protein. These data substantiate the hypothesis that etoposide preferentially targets topo IIα, while amsacrine targets topo IIβ. In addition we have shown that we can successfully manipulate topo IIα gene expression in cells without the problem of feedback inhibition previously experienced by us and other laboratories (Asano et al, 1996). We attributed this to our use of the strong cytomegalovirus promoter in our adenoviral vector construct. This vector can be manipulated by making mutations in specific parts of the gene and thus provide a valuable tool with which to investigate the biology of human topo IIα expression and function.

### ACKNOWLEDGEMENTS

We wish to thank Dr L Liv, Robert Wood Johnson Medical School,

 *© 2001 Cancer Research Campaign*

BJOC 01-1966 747-751 20/8/01 3:29 pm Page 751

University of Medicine and Dentistry of New Jersey, Piscataway, NJ, USA for the generous gift of human topo IIα gene probe ZII69. We also wish to thank Dr Caroline Austin, University of Newcastle-upon-Tyne, Newcastle, UK for the gift of topo IIβ gene probe F12. This work was supported in part by NIH Grants CA42992 (to ESK), CA40090 (to LAZ), and Cancer Center Support Core Grant CA16672 (to MD Anderson Cancer Center).

### REFERENCES

Asano T, An T, Mayes J, Zwelling LA and Kleinerman ES (1996) Transfection of human topoisomerase IIα gene into etoposide-resistant cells: transient increase in sensitivity followed by down-regulation of the endogenous gene. *Biochem J* **319**: 307–313

Asano T, An T, Zwelling LA, Takano H, Fojo AT and Kleinerman ES (1996) Transfection of a human topoisomerase IIα gene into etoposide-resistant human breast tumor cells sensitizes the cell to etoposide. *Oncol Res* **8**: 101–110

Austin CA and Marsh KL (1998) Eukaryotic DNA topoisomerase IIβ. *Bioessays* **20**: 215–226

Austin CA, Song J, Patel S and Fisher LM (1993) Novel Hela topoisomerase is the IIβ isoform: complete coding sequence and homology with other type II topoisomerases. *Biochim Biophys Acta* **172**: 283–291

Dereuddre S, Delaporte C and Jacquemin-Sablon A (1997) Role of topoisomerase II beta in the resistance of 9-OH-ellipticine-resistant Chinese hamster fibroblast to topoisomerase II inhibitors. *Cancer Res* **57**: 4301–4308

Errington F, Willmore E, Tilby MJ, Li L, Li G, Baguley BC and Austin CA (1999) Murine transgenic cells lacking DNA topoisomerase IIb are resistant to Acridines and Mitoxantrone: Analysis of cytotoxicity and cleavable complex formation. *Mol Pharmacol* **56**: 1309–1316

Fan D, Poste G, Ruffoli RR, Dong Z, Seid C, Earnest LE, Campbell TE, Clyne RK, Beltran PJ and Fidler IJ (1994) Circumvention of multidrug resistance in murine fibrosarcoma and colon carcinoma cells by treatment with the α-adrenoceptor antagonist furobenzepine. *Int J Oncol* **4**: 789–798

Ganapathi R, Constantinou A, Kamath N, Dubyak G, Grabowski D and Krivacic K (1996) Resistance to etoposide in human leukemia HL-60 cells: Reduction in drug-induced DNA cleavage associated with hypophosphorylation of topoisomerase II phosphopeptides. *Mol Pharmacol* **50**: 243–248

Herzog CE, Holmes KA, Tuschong LM, Ganapathi R and Zwelling LA (1998) Absence of topoisomerase IIβ in an amsacrine-resistant human leukemia cell line with mutant topoisomerase IIα. *Cancer Res* **58**: 5298–5300

Jenkins JR, Ayton P, Jones T, Davies SL, Simmons DL, Harris AL, Sheer D and Hickson ID (1992) Isolation of cDNA clones encoding the β isozyme of human DNA topoisomerase II and localization of the gene to chromosome 3q24. *Nucleic Acids Res* **20**: 5587–5592

Koo H-M, Gray-Goodrich M, McWilliams MJ, Jeffers M, Vaigro-Wolff A, Alvord WG, Monks A, Paull KD, Pommier Y and Woude GFV (1999) The ras omcogene-mediated sensitization of human cells to toposiomerase II inhibitor-induced apoptosis. *J Natl Cancer Inst* **91**: 236–244

Malonne H and Atassi G (1997) DNA topoisomerase targeting drugs: mechanism of action and perspectives. *Anticancer Drugs* **8**: 811–822

Matsumoto Y, Takano H and Fojo T (1997) Cellular adaptation to drug exposure: evolution of the drug-resistant phenotype. *Cancer Res* **57**: 5086–5092

Tan KB, Dorman TE, Falls KM, Chung TD, Mirabell CK, Crooke ST and Mao J (1992) Topoisomerase IIα and topoisomerase IIβ genes: Characterization and mapping to human chromosome 17 and 3, respectively. *Cancer Res* **52**: 231–234

Tsai-Pflugfeder M, Liu LF, Liu AA, Tewey KM, Whang-Peng J, Knutsen T, Huebner K, Croce CM and Wang JC (1988) Cloning and sequencing of cDNA encoding human DNA topoisomerase II and localization of the gene to chromosome region 17q21–22. *Proc Natl Acad Sci USA* **85**: 7177–7181

Turley H, Comley M, Houlbrook S, Nozaki N, Kikuchi A, Hickson ID, Gatter K and Harris AL (1997) The distribution and expression of the two isoforms of DNA topoisomerase II in normal and neoplastic human tissues. *Br J Cancer* **75**: 1340–1346

Wang JC (1996) DNA topoisomerase. *Annu Rev Biochem* **65**: 635–692

Withoff S, Vries E, Keith WN, Nienhuis EF, Graaf WTA, Uges DRA and Mulder NH (1996) Differential expression of DNA topoisomerase II alpha and -beta in P-gp and MRP-negative VM26, mAMSA and mitoxantrone-resistant sublines of the human SCLC cell line $GLC_4$. *Br J Cancer* **74**: 1869–1876

Yang X, Li W, Prescott ED, Burden SJ and Wang JC. (2000) DNA topoisomerase IIβ and neural development. *Science* **287**: 131–134

Zwelling LA, Hinds M, Chan D, Mayers J, Sie KL, Parker E, Silberman L, Radcliffe A, Beran M and Blick M (1989) Characterization of an amsacrine-resistant line of human leukemia cells. *J Biol Chem* **264**: 16411–16420

Zhou Z, Zwelling LA, Kawakami Y, An T, Kobayahi K, Herzog C and Kleinerman ES (1999) Adenovirus-mediated human topoisomerase IIα gene transfer increases the sensitivity of etoposide-resistant human breast cancer cells.

© 2001 Cancer Research Campaign
